# 5‐ALA Assisted Surgery of Human Glioblastoma Samples Reveals an Enrichment of T Cells Expressing PD‐1 and CD103 in the Intermediate and Marginal Layers

**DOI:** 10.1002/eji.202451681

**Published:** 2025-06-11

**Authors:** Anna Vanni, Francesca Matani, Camilla Bonaudo, Alessio Mazzoni, Manuela Capone, Giulia Lamacchia, Lorenzo Salvati, Lucia Bartoli, Stefania Francalanci, Mirko Petti, Federico Capelli, Filippo Nozzoli, Lorenzo Cosmi, Francesco Liotta, Alessandro Della Puppa, Laura Maggi, Francesco Annunziato

**Affiliations:** ^1^ Department of Experimental and Clinical Medicine University of Florence Florence Italy; ^2^ Neurosurgery Department of NEUROFARBA University of Florence University Hospital of Careggi Florence Italy; ^3^ Flow Cytometry Diagnostic Center and Immunotherapy Careggi University Hospital Florence Italy; ^4^ Department of Laboratory Medicine Azienda USL‐Toscana Centro Florence Italy; ^5^ Section of Anatomic Pathology Department of Health Sciences University of Florence Florence Italy; ^6^ Immunoallergology Unit Careggi University Hospital Florence Italy; ^7^ Immunology and Cell Therapy Unit Careggi University Hospital Florence Italy

**Keywords:** glioblastoma, 5‐ALA, T cells, immune checkpoints, immunotherapy

## Abstract

Glioblastoma is the most common malignant brain tumor in adults, for which immunotherapy shows reduced efficacy. Current knowledge on immunotherapy failure is limited and detailed information about immune infiltrates in glioblastoma is urgently needed. We enrolled 34 glioblastoma patients collecting peripheral blood (PB), total tumor resection, or tumor from the necrotic area, the intermediate, and the marginal tissue through 5‐aminolevulinic‐acid (5‐ALA) assisted surgery. T cells were evaluated for immune checkpoints and tissue residence memory (Trm) cell markers expression, and their cytokine production profile. Biological data were correlated with the patient's overall survival. Flow cytometry analysis showed a significantly higher frequency of T lymphocytes expressing PD‐1, Trm markers in glioblastoma than in PB. In particular, we observed a preferential enrichment of CD8 cells expressing PD‐1 and Trm markers in the intermediate and marginal tissue. T cells cytokine production resulted in increased glioblastoma compared with PB, in particular in PD‐1+ cells and in the intermediate and marginal layers. These data suggest that CD103+ T‐cell frequency in the core and TNF‐a+CD8+ T cells in the intermediate layer influence the patient's survival. In conclusion, T cells obtained from different GBM layers showed different phenotypes and cytokines expression, suggesting new prognostic factors and supporting surgery particle strategy.

## Graphical abstract

Flow‐cytometric analysis of glioblastoma immune infiltrates showed a significantly higher frequency of T lymphocytes expressing PD‐1, CD69, and CD103. In particular, we observed a preferential enrichment of CD8+ T cells expressing PD‐1 and Trm markers in intermediate and marginal tumor areas, in which glioblastoma cells showed an active 5‐ALA metabolism.

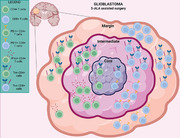

## 1 Background

Glioblastoma (GBM, astrocytoma, WHO grade IV) is the most common and deadly primary brain tumor with a median survival of less than 1 year. The poor clinical outcome of patients with GBM underscores the urgent need for effective therapies outside of the traditional approaches including surgical resection, chemotherapy, and radiation therapy [1, 2].

In many malignancies, immunotherapy with PD‐1/PD‐L1 pathway inhibition has arisen as a successful strategy [3]. Unfortunately, clinical trials targeting PD‐1/PD‐L1 in GBM patients failed to improve progression‐free or overall survival [4, 5]. The challenges of immunotherapy to treat GBM are numerous such as a limited understanding of basic cellular mechanisms governing anti‐tumor responses in the brain, the peculiar tumor location that could obstacle therapy delivery, in addition to a complex and suppressive tumor microenvironment (TME): GBM secretion of chemokines, cytokines and growth factors promotes infiltration of immunosuppressive immune cells, such as macrophages, myeloid‐derived suppressor cells (MDSC) and Treg cells [6] and a severely exhausted T‐cell tumor infiltrate [7]. However, current knowledge on the biological mechanisms responsible for immunotherapy failure in GBM is limited [8, 9, 10] and detailed information about the composition of immune cell infiltrates in GBM are urgently needed to personalize antitumor therapeutic strategy.

Fluorescence‐guided surgery using 5‐aminolevulinic acid (5‐ALA) has been introduced in the treatment of malignant gliomas [11]. Through this technique, it is possible to identify different areas of GBM with different metabolic features and perform a tumor resection more accurately than with white light surgery. Depending on their location in the TME, different immune cells may play a specific role in anti‐tumor response and influence GBM development. In fact, it has already been described that bone marrow‐derived macrophages (BMDM) compared with resident Microglia have an increasing concentration and immunosuppressive activity, moving from the periphery to the center of the lesion [12]. The application of 5 ALA‐guided surgery is an interesting approach to obtaining fresh tumor specimens that allow the localized analysis of immune cells in TME in association with the different tumor areas.

In the present study, we performed a phenotypical and functional characterization of tumor‐infiltrating T cells evaluating tissue resident markers, the inhibitory immune‐checkpoints (ICs) TIGIT and PD‐1 and correlating ICs expression with cytokine production capacity in GBM patients, analyzing how T cells phenotype changes within different tumor areas with different metabolic features.

## 2 Materials and Methods

### 2.1 Patients

In this study, we enrolled 34 patients over the age of 18 with a radiological diagnosis of high‐grade glioma operated by the same senior neurosurgeon in the Neurosurgical Department, at the University Hospital of Florence (Azienda Ospedaliera Universitaria Careggi, AOUC), Italy. All of them underwent a gross total resection, with the use of intraoperative monitoring, tractography, and the neuro‐navigation system, respecting the principle of the onco‐functional balance [13]. Among them, 17 were operated in white light (W‐L), while 17 were operated with the use of 5‐ALA to allow the stratification in three different tumor layers: the central necrotic area (core), corresponding to the inner non‐fluorescent tissue, an intermediate area, brightly fluorescent (intermediate), and a marginal area corresponding to a dimly fluorescent tissue (margin). In detail, 5‐ALA fluorescence‐collected GBM biopsies could be related to different preoperative MRI sequences and histological features: inner non‐fluorescent tissue (core) corresponds to the non‐contrast enhancing MRI sequence and to the tumor necrosis; the bright fluorescent (intermediate) area corresponds to the contrast‐enhancing MRI sequence and the tumor; dimly fluorescent (margin) area corresponds to peritumoral infiltration, visible in MRI FLAIR sequences. The main clinical features of recruited patients are shown in Table S1.

### 2.2 Collection of PBMCs

Peripheral blood (PB) was drawn from patients prior to surgical tumor resection and, if the case, prior to 5‐ALA administration. PB mononuclear cells (PBMCs) were obtained following density gradient centrifugation of samples using Lymphoprep (Axis‐Shield Poc As). PBMCs were then stained or stimulated for subsequent Flow Cytometry evaluation along with paired Tumor Infiltrated Lymphocytes (TILs).

### 2.3 Collection of TILs

Fresh tumor samples were processed within 4 h for immune infiltrate isolation. Tumor samples obtained from total GBM by white‐light (W‐L) resection or from 5‐ALA‐assisted surgery, underwent mechanical fragmentation and then to enzymatic digestion with collagenase D (Sigma Aldrich) at a concentration of 1 mg/mL for 15 min. After the digestion step, the homogenized tissue was filtered to obtain a single‐cell suspension. Cell pellets were then resuspended in PBS and then stratified on percoll 30% for myelin elimination and TILs isolation. TILs were then washed twice in RPMI 10% FCS and directly stained or stimulated for subsequent Flow Cytometry analysis.

### 2.4 Immunophenotyping by Flow Cytometry

For lymphocyte cell subsets analysis by surface markers expression, PBMCs, and TILs, obtained from total GBM by W‐L resection or GBM layers by 5‐ALA assisted surgery, were washed in PBS+BSA 0,5% and then stained for 15 min with fluorochrome‐conjugated monoclonal antibodies (mAbs), using the panels described in Table S2. The gating strategy is shown in Figure S1, samples were acquired on a BD LSR II flow cytometer (BD Biosciences).

### 2.5 Intracellular Cytokine Production Assay by Flow Cytometry

PBMCs and TILs, obtained from total GBM by W‐L resection or GBM layers by 5‐ALA assisted surgery, were polyclonally stimulated for 5 h with PMA (10 ng/mL) and Ionomycin (1 µM) for 5 h, the last 3 in the presence of Brefeldin A (5 µg/mL). Cells were then fixed in formaldehyde 2% (15 min at RT), washed in PBS+BSA 0.5% and then stained intracellularly with fluorochrome‐conjugated mAbs using panels described in Table S3 (PBMCs and total GBM resection) and Table S4 (PBMCs and GBM layers resected with 5‐ALA assisted surgery), in a buffer containing the permeabilizing agent Saponin (0.5%). Samples were acquired on a BD LSR II flow cytometer (BD Biosciences). The gating strategies are shown in Figures S2 and S3. All flow cytometric analyses were performed following published guidelines [14].

### 2.6 Statistical Analysis

The statistical evaluation of differences between conditions or groups was performed by the Mann–Whitney *U* test or the Wilcoxon test for paired samples. Regarding the statistical evaluation of differences in the tumor layers of the 5‐ALA cohort (core, intermediate, and margin), it was performed using Friedman ANOVA, followed by the Wilcoxon test when statistically significant. Pearson's correlation coefficients were used to calculate the correlations between parameters. *p*‐values less than 0.05 were considered significant.

## 3 Results

### 3.1 CD8+ Tumor Infiltrating Lymphocytes Are Enriched in the GBM Marginal Area

**FIGURE 1:**
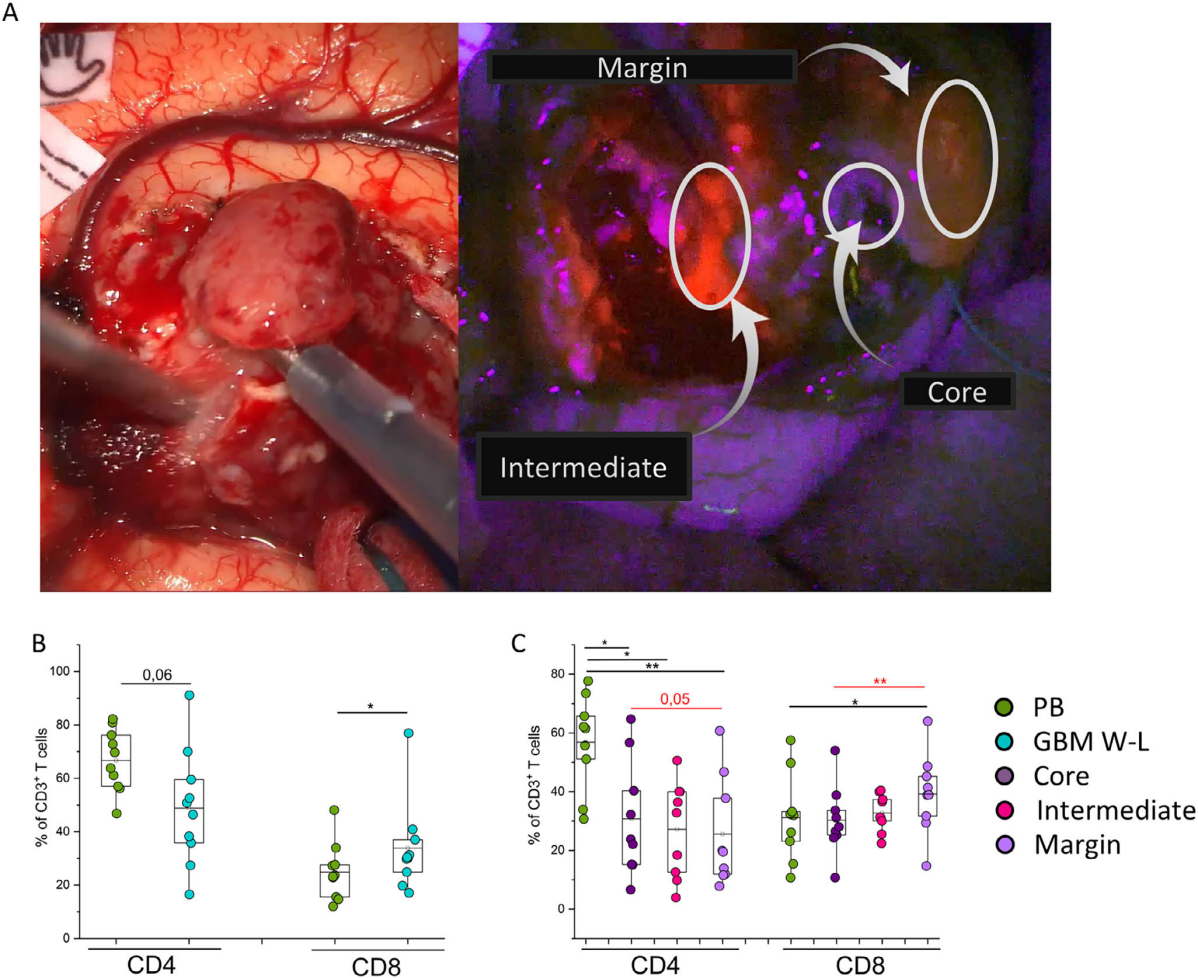
Evaluation of CD4 and CD8 percentage among CD3+ T lymphocytes obtained from PB, total W‐L GBM, or different tumor areas identified by 5‐ALA fluorescence. (A) Representative images of the same tumor, under white light (showing a pathological tissue) and under blue light with the intraoperative use of the blue filter directly applied to the intraoperative microscope (Zeiss Kinevo 70). With a blue filter, it is possible to identify 5‐ALA tumor layers: non‐fluorescent core, bright fluorescent, and vague fluorescent periphery. (B) Percentage of CD4+ and CD8+ T cells in PB (green dots, *n* = 9) or total W‐L GBM resection (blue dots, *n* = 9). (C) Percentage of CD4+ and CD8+ T cells in PB (green dots, *n* = 9) or tumor areas identified by 5‐ALA florescence‐assisted surgery, in particular, core (dark‐purple dots, *n* = 9), intense (pink dots, *n* = 9), and margin (light‐violet dots, *n* = 9). Boxes indicate mean values and 25th and 75th percentiles. Whiskers indicate minimum and maximum values. Statistical analysis was performed by Friedman ANOVA and Wilcoxon‐signed rank test (*p < 0.05, **p < 0.01). Black asterisks refer to paired statistics of PB compared with tumor samples. Red asterisks represent paired statistics among tumor layers identified based on 5‐ALA‐assisted surgery.

To gain insight into possible mechanisms of tumor escape and recurrence in gliomas we phenotypically characterized infiltrating T lymphocytes in Glioblastoma. We first evaluated the frequencies of CD4+ and CD8+ T cells using flow cytometry, calculated as percentage of CD3+ T cells among TILs from tumor samples resected in white light (W‐L GBM) or from tumor samples resected with 5‐ALA‐assisted surgery and from PMNC of nine paired patients for each cohort. Following preoperative administration of 5‐ALA, fluorescent protoporphyrin IX (PpIX) is synthesized and can be visualized under violet light with different fluorescence intensities, allowing the identification of three layers within the tumor mass: the central necrotic area (core), an intermediate brightly fluorescent area (intermediate), and dimly fluorescent marginal area (margin) (Figure 1A).

In W‐L GMB samples compared with PB, there was a lower frequency of CD4+ T cells and a statistically significant higher frequency of CD8+ T cells (Figure 1B). This inversion of CD4+ and CD8+ T‐cell frequency in tumors compared with PB samples is mainly related to the marginal tumor area, in fact, we observed a significantly lower CD4+ T‐cell frequency in the margin compared with PB, while the intense and core layers displayed frequencies similar to PB. On the other side, CD8+ T cells were significantly higher in the margin compared with both PB and tumor core (Figure 1C).

### 3.2 GBM TILs Expressing High Levels of Trm Markers and PD‐1 Are Preferentially Enriched in the Intermediate and Marginal Tumor Areas

**FIGURE 2:**
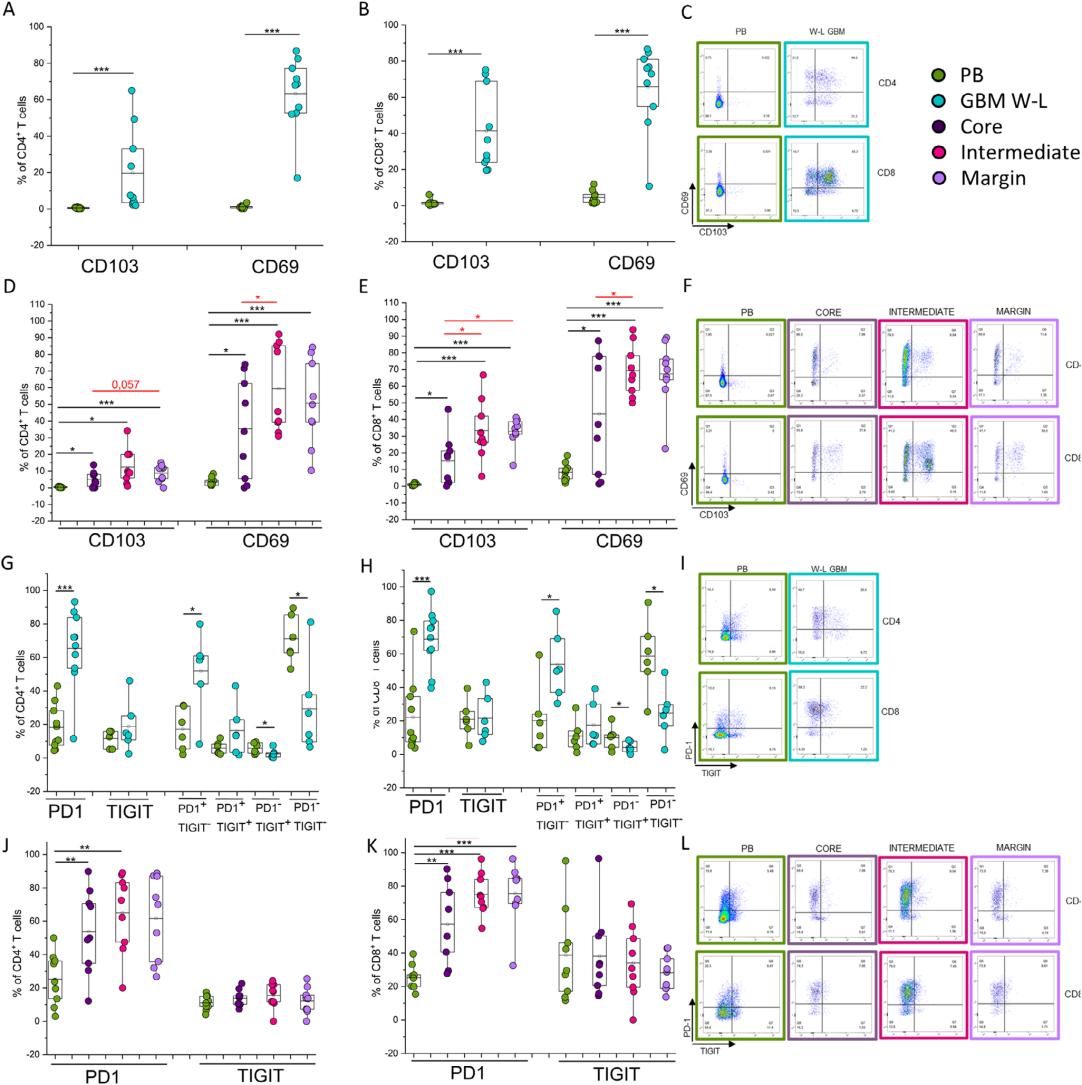
Evaluation of Trm markers and inhibitory immune‐checkpoint expression in T lymphocytes obtained from PB, total W‐L GBM, or different tumor areas identified based on 5‐ALA fluorescence. (A) Percentage of CD4+ and (B) of CD8+ T cells expressing the Trm markers CD103 and CD69 obtained from PB (green dots, *n* = 9) or W‐L GBM resection (blue dots, *n* = 9). (C) Representative plots of CD4+ and CD8+ T cells expressing CD103 and CD69 were obtained from PB or W‐L GBM. (D) Percentage of CD4+ and (E) of CD8+ T cells expressing the Trm markers CD103 and CD69 obtained from PB (green dots, *n* = 9) or tumor areas identified based on 5‐ALA assisted surgery, in particular core (dark‐purple, dots *n* = 9), intense (pink dots, *n* = 9), and margin (light‐violet dots, *n* = 9). (F) Representative plots of CD4+ and CD8+ T cells expressing CD103 and CD69 were obtained from PB or tumor areas identified based on 5‐ALA‐assisted surgery. (G) Percentage of CD4+ and (H) of CD8+ T cells expressing the ICs PD‐1 (*n* = 9), TIGIT (*n* = 6) or the combinatorial expression (*n* = 6) of both markers (PD‐1+TIGIT−, PD‐1+TIGIT+, PD‐1−TIGIT+, PD‐1−TIGIT−) obtained from PB (green dots) or total W‐L GBM resection (blue dots). (I) Representative plot of CD4+ and CD8+ T cells expressing PD‐1 and TIGIT obtained from PB or W‐L GBM. (J) Percentage of CD4+ and (K) of CD8+ T cells expressing the ICs PD‐1, TIGIT obtained from PB (green dots, *n* = 9) or tumor areas identified based on 5‐ALA‐assisted surgery, in particular, core (dark‐purple dots, *n* = 9), intense (pink dots, *n* = 9), and margin (light‐violet dots, *n* = 9). (L) Representative plots of CD4+ and CD8+ T cells expressing PD‐1 and TIGIT obtained from PB or tumor areas identified based on 5‐ALA assisted surgery. Boxes indicate mean values and 25th and 75th percentiles. Whiskers indicate minimum and maximum values. Statistical analysis was performed by Friedman ANOVA and Wilcoxon‐signed rank test (*p < 0.05, **p < 0.01, ***p < 0.001). Black asterisks refer to paired statistics of PB compared with tumor samples. Red asterisks represent paired statistics among tumor layers identified based on 5‐ALA‐assisted surgery.

We also evaluated the expression of two tissue‐residency markers (CD69 and CD103), which identify memory T lymphocytes permanently stationing in the tissue, Trm cells involved in providing “in situ” immunity. Accordingly, the frequencies of CD69 and CD103 expressing cells were dramatically higher in both CD4+ and CD8+ T cells’ population from tumor samples than from PB, both in the case of W‐L GBM (Figure 2A–C) and in the case of all the three layers of 5‐ALA resected GBM, compared with PB (Figure 2D–F). Interestingly, in tumor samples obtained with the 5‐ALA technique, we observed an enrichment of the frequency of CD4+T cells expressing the Trm markers CD69 in the intermediate, metabolic active, tumor area than in core tissues (Figure 2D), whereas no difference was observed regarding the frequency of CD103 expressing CD4+ T cells (Figure 2D). Similar results were obtained for CD8+ T cells although with a significant increase of the frequency of T cells expressing CD69 and CD103 in both the marginal and intermediate area compared with the necrotic core (Figure 2E).

To understand whether infiltrating T cells enriched in Trm markers showed signs of exhaustion we investigated by flow cytometry the expression of two distinctive immune checkpoints (ICs), PD‐1 and TIGIT. We observed that W‐L GBM samples were significantly enriched in CD4+ and CD8+ T cells expressing PD‐1 when compared with PB (Figure 2G–I), whereas the frequency of TIGIT expressing CD4 and CD8 T cells was comparable in PB and tumors (Figure 2G–I). Of note, the combinatorial expression of the two ICs defined four distinct subsets: PD‐1+TIGIT−, PD‐1+TIGIT+, PD‐1−TIGIT+, PD‐1−TIGIT−. CD4+ and CD8+ T cells with a PD‐1−TIGIT− and a PD‐1−TIGIT+ phenotype were significantly enriched in PB than GBM samples. On the contrary, the frequency of both CD4+ and CD8+ T‐cell subsets, PD‐1+TIGIT– were significantly higher in GBM than in PB (Figure 2G,H).

Analyzing PD‐1 and TIGIT expression on CD4+ T cells on samples from 5‐ALA assisted surgery, we observed a higher percentage of PD‐1+ T cells in all analyzed different tumor areas than in PB, whereas no difference was observed for TIGIT and for both ICs expression among the three different GBM layers (Figure 2J,L). Similarly, for CD8+ T cells we observed a higher frequency of PD1+CD8+ T cells in samples from all tumor areas compared with PB, but in this case, the highest proportion of cells expressing PD‐1 was detected in the intense and marginal area (Figure 2K,L). Regarding the distribution of CD8+ T cells expressing TIGIT, no relevant difference was observed in all analyzed samples (Figure 2K).

### 3.3 GBM TILs Express High Level of Proinflammatory Cytokines in Particular the PD‐1 Positive Subpopulation

To evaluate the effector function of total and IC‐expressing CD4+ and CD8+ T‐cell subsets, we assessed their expression levels of proinflammatory cytokines (IFN‐γ, TNF‐α, GM‐CSF, IL‐17) and anti‐inflammatory cytokine IL‐10, in both PB and WL‐GBM samples.

**FIGURE 3:**
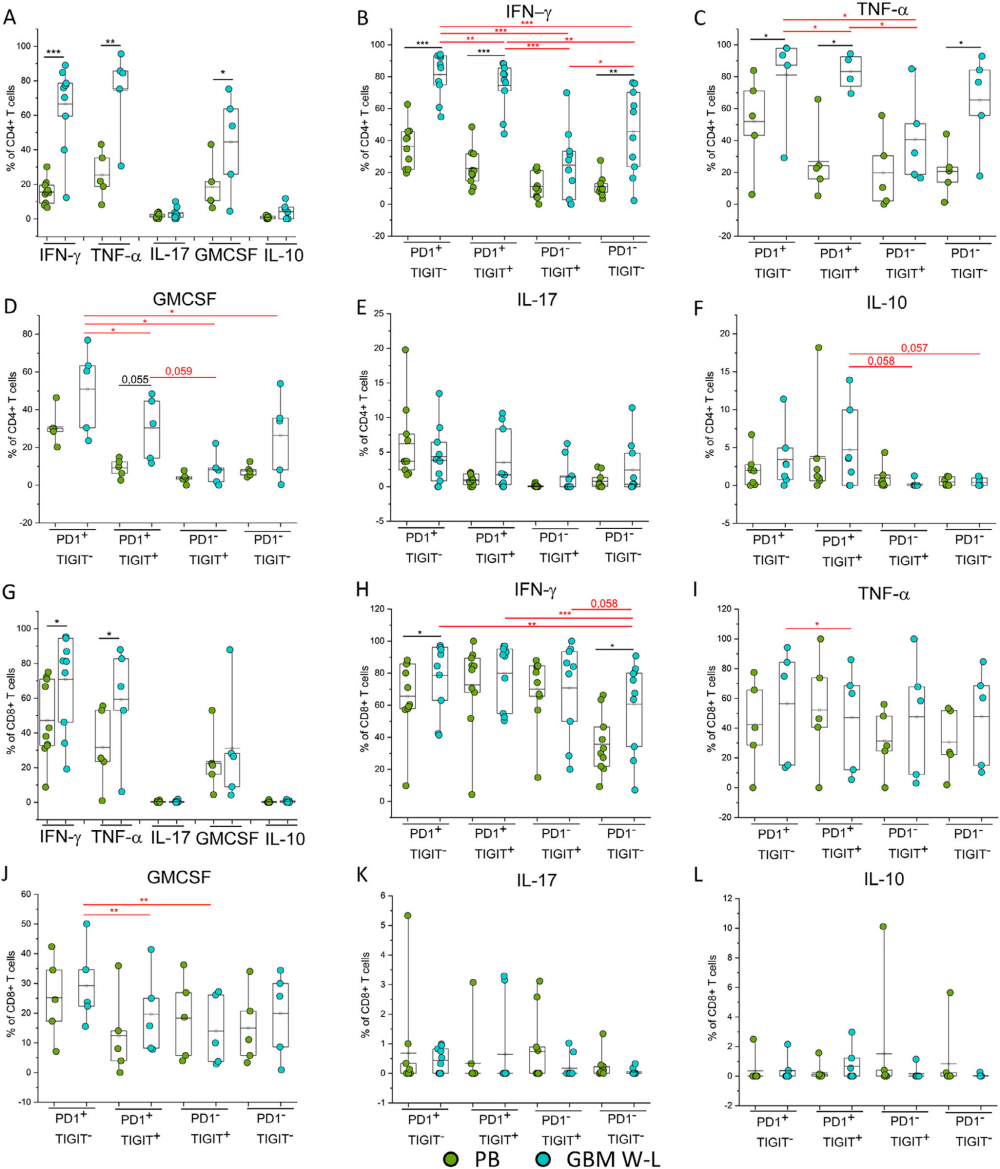
Cytokine production in PB and W‐L GBM CD4+ and CD8+ T cell subsets expressing distinct combinations of inhibitory immune checkpoints. (A) Frequency of total CD4+ T cells producing: IFN‐γ (*n* = 10), TNF‐α (*n* = 5), IL‐17 (*n* = 9), GM‐CSF (*n* = 5), and IL‐10 (*n* = 7). Frequency of CD4+ T cells subsets (PD‐1+TIGIT−, PD‐1+TIGIT+, PD‐1−TIGIT+, PD‐1−TIGIT−) producing IFN‐γ (B), TNF‐α (C), GM‐CSF (D), IL‐17 (E), and IL‐10 (F). (G) Frequency of total CD8+ T cells producing: IFN‐γ (*n* = 10), TNF‐α (*n* = 5), IL‐17 (*n* = 9), GM‐CSF (*n* = 5), and IL‐10 (*n* = 7). Frequency of CD8+ T‐cell subsets (PD‐1+TIGIT−, PD‐1+TIGIT+, PD‐1−TIGIT+, PD‐1−TIGIT−) producing IFN‐γ (H), TNF‐α (I), GM‐CSF (J), IL‐17 (K), IL‐10 (L). (A–L) T cells were obtained from PB (green dots) and total WL‐GBM resection (blue dots) samples. Boxes indicate mean values and 25th and 75th percentiles. Whiskers indicate minimum and maximum values. Statistical analysis was performed by Wilcoxon‐signed rank test (*p < 0.05, **p < 0.01, ***p < 0.001). Black asterisks refer to paired statistics of PB compared with tumor samples. Red asterisks represent paired statistics among subsets in GBM samples defined by TIGIT/PD‐1 expression.

As shown in Figure 3A, CD4+ T cells from WL‐GBM expressed, following ex‐vivo polyclonal stimulation, significantly higher levels of the proinflammatory cytokines IFN‐γ, TNF‐α, and GM‐CSF compared with PB. On the contrary, no differences were observed in terms of IL‐17 and IL‐10 expression between the two tissues.

Focusing on cytokine production by the four subsets identified by TIGIT and PD‐1 expression (Figure 3B–F), we observed a higher frequency of CD4+ T cells expressing the proinflammatory cytokines IFN‐γ, TNF‐α, and GM‐CSF in WL‐GBM samples compared with the corresponding PB populations with statistical significance for IFN‐γ and TNF‐α expression in the PD‐1+ TIGIT−, PD‐1+TIGIT+ and PD‐1−TIGIT− subpopulations (Figure 3B–D). According to data on total CD4+ T cells (Figure 3A), the frequency of cells expressing IL‐17 and IL‐10 was comparable between the four different subpopulations in PB and GBM samples. Analyzing GBM infiltrating CD4+ T cells, we noticed a hierarchy in cytokine production capability by the four subsets defined by PD‐1 and TIGIT expression. Indeed, the higher percentage of cells producing higher levels of all proinflammatory cytokines (IFN‐γ, TNF‐α, GM‐CSF) were observed in the PD‐1+ TIGIT‐ subset followed by the PD‐1+TIGIT+ and then PD‐1−TIGIT− populations. The PD‐1−TIGIT+ population instead exhibited the lowest percentage of cells expressing these cytokines (Figure 3B–D). Regarding the frequency of cells expressing the anti‐inflammatory cytokine IL‐10, there was a not statistically significant tendency to be higher in the PD‐1+TIGIT+ populations (Figure 3F), whereas no difference was observed in IL‐17 expression among the four subpopulations (Figure 3E).

Similar to CD4+ T lymphocytes, also in CD8+ T cells we observed a significantly higher expression of the proinflammatory cytokines IFN‐γ and TNF‐α in GBM compared with PB (Figure 3G). GM‐CSF, IL‐17, and IL‐10 were instead comparable between the two tissues. On the contrary, CD8+ T cells were characterized by a different feature from CD4+ ones, when we focused on the four subsets, identified by TIGIT and PD‐1 expression (Figure 3H–L): their ability to produce the analyzed cytokines was comparable between GBM and PB samples in all the four subpopulations with the exception of the frequency of IFN‐γ expressing cells in the PD‐1+ TIGIT– and PD‐1− TIGIT– subsets which were higher in GBM compared with PB (Figure 3H). Among GBM infiltrating CD8+ T cells, the PD‐1+TIGIT– subpopulation was characterized by the significantly higher frequency of cells expressing IFN‐γ, TNF‐α, and GM‐CSF, followed by the other three subpopulations (Figure 3H–J), while no difference was observed in IL‐17 and IL‐10 production (Figure 3K,L).

### 3.4 CD4+ T Cells Infiltrating the Intermediate Tumor Area Showed High Expression of the Proinflammatory Cytokine IFN‐γ

We had the opportunity to study the cytokine production profile of TILs in the three tumor layers obtained with 5‐ALA‐assisted surgery on an additional seven patients, after ex vivo polyclonal stimulation.

**FIGURE 4:**
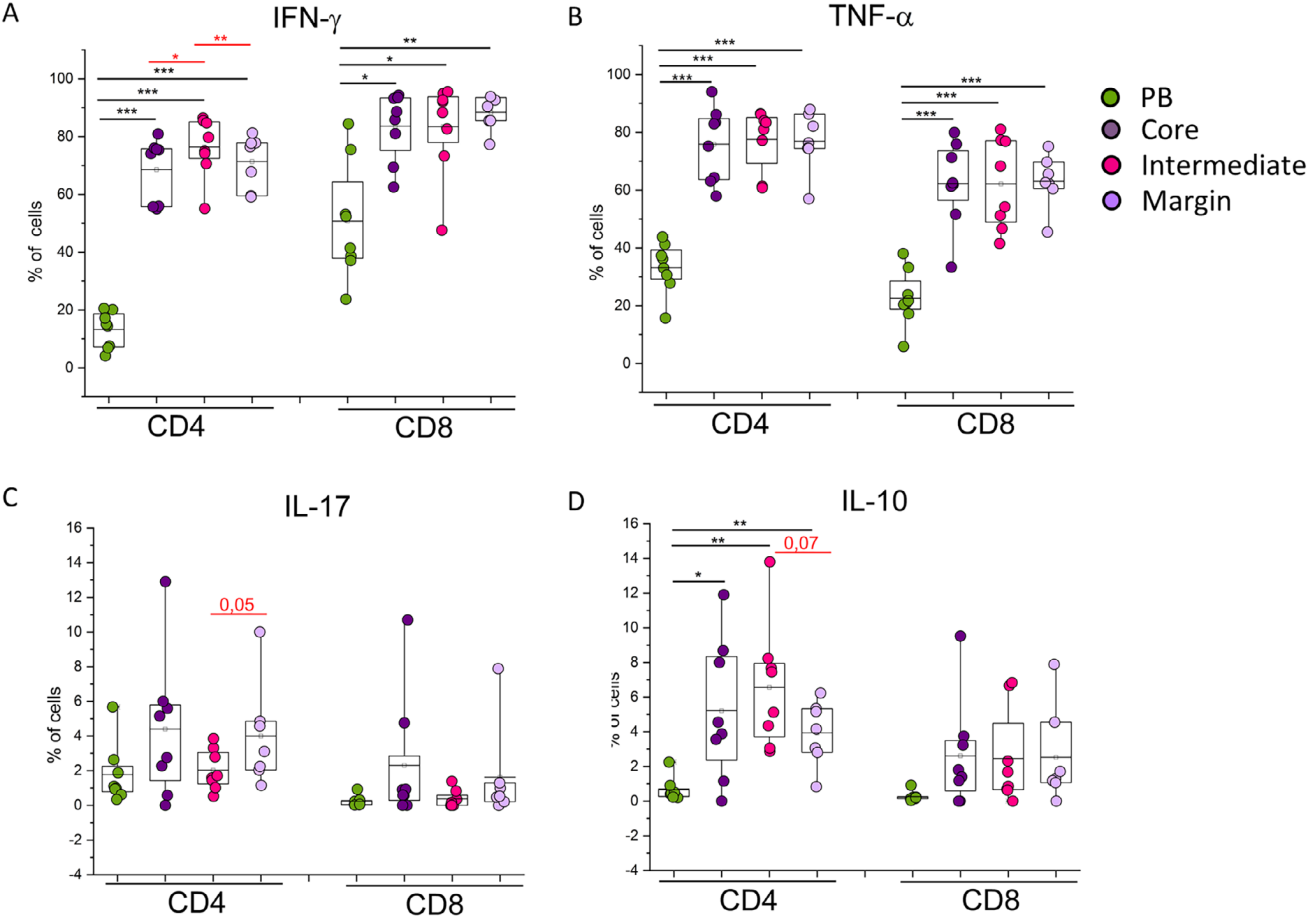
Cytokine production in GBM CD4+ and CD8+ T cells from different tumor layers. Frequency of total CD4+ and CD8+ T cells producing (A) IFN‐γ, (B) TNF‐α (C) IL‐17, and (D) IL‐10 obtained from PB (green dots, *n* = 7) or tumor areas identified based on 5‐ALA assisted surgery, in particular core (dark‐purple dots, *n* = 7), intense (pink dots, *n* = 7), and margin (light‐violet dots, *n* = 7). Boxes indicate mean values and 25th and 75th percentiles. Whiskers indicate minimum and maximum values. Statistical analysis was performed by Friedman ANOVA and Wilcoxon‐signed rank test (*p < 0.05, **p < 0.01, ***p < 0.001). Black asterisks refer to paired statistics of PB compared with tumor samples. Red asterisks represent paired statistics among tumor layers identified based on 5‐ALA‐assisted surgery.

For both CD4+ and CD8+ T‐cell subsets, we observed a significantly higher frequency of IFN‐γ and TNF‐α expressing cells in the three GBM layers compared with PB (Figure 4A,B), whereas no difference was observed for IL‐17 in all analyzed samples (Figure 4C). Interestingly, in the CD4+ T‐cell subset, also the frequency of cells expressing the anti‐inflammatory cytokine IL‐10 was significantly higher in the three tumor layers compared with PB (Figure 4D). Among the three different tumor areas, we observed a significantly higher frequency of CD4+ T cells expressing IFN‐γ and a slight but not statistically significantly lower expression of IL‐17, in the intermediate tumor area compared with the core and marginal area (Figure 4A,C), whereas no difference was observed for TNF‐α and IL‐10 and (Figure 4 B,D). Considering CD8+ T‐cell cytokine production, no relevant difference was observed among the three different tumor layers for the analyzed cytokines.

### 3.5 Frequency of CD103+ T Cells and CD8+TNF‐α+ T Cells Showed Respectively a Positive and a Negative Correlation With Patient's Overall Survival

**FIGURE 5:**
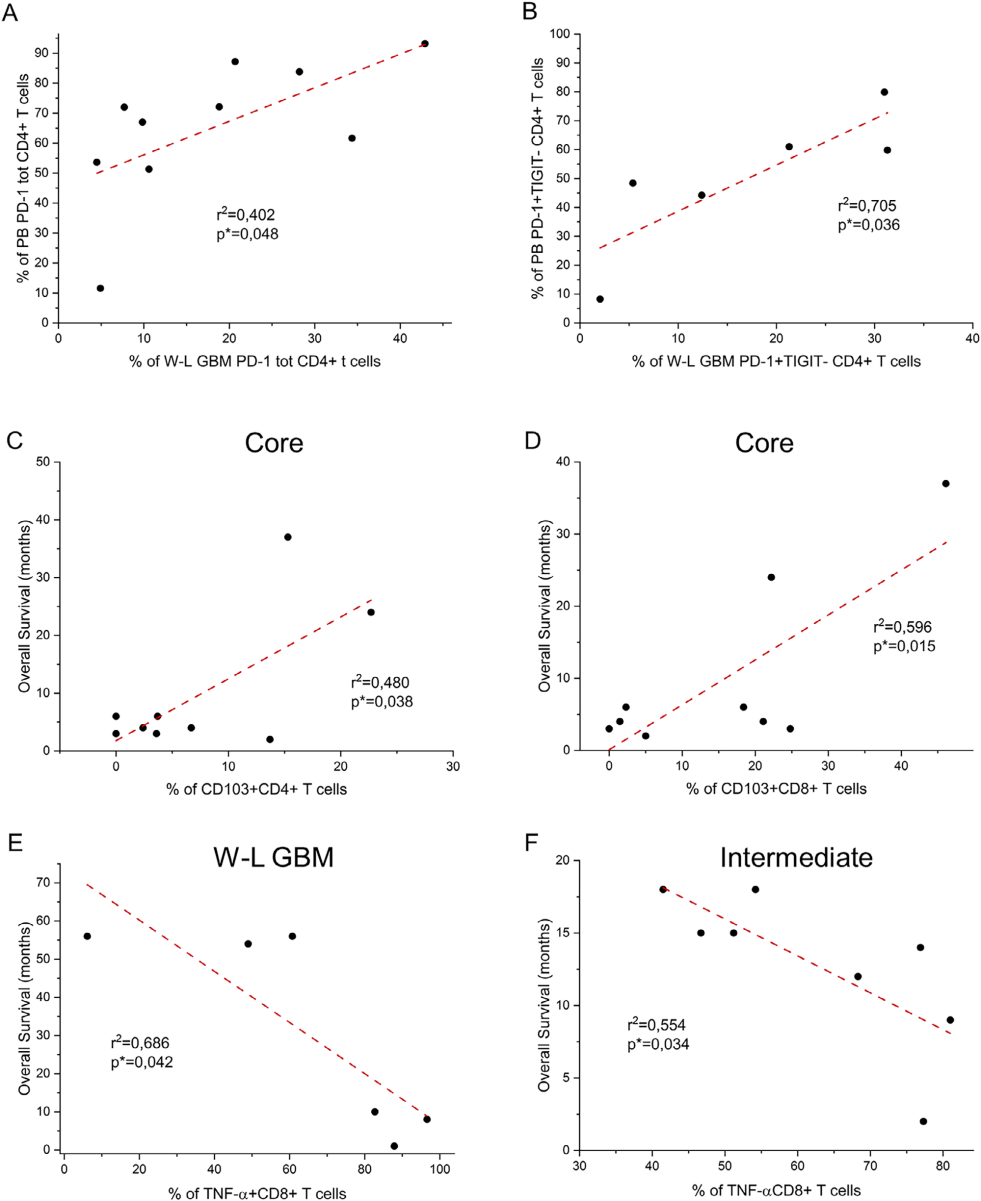
Correlation between ICs, Trm markers, and cytokines expression in GBM infiltrating CD4+ and CD8+ T cells and patients’ clinical parameters. (A) Correlation between the percentage of CD4+ T cells expressing PD‐1 in PB and W‐L GBM (*n* = 10). (B) Correlation between the percentage of PD‐1 single positive (PD‐1+TIGIT−) CD4+ T cells in PB and in W‐L GBM (*n* = 6). Correlation between the percentage of CD103 positive CD4+ (*n* = 9) (C) or CD8+ (*n* = 9) (D) T cells, obtained from the necrotic core, and patients’ overall survival. Correlation between the percentage of TNF‐positive CD8+ T cells and patients’ overall survival in W‐L GBM (*n* = 6) (E) or in the intermediate layer (*n* = 8) (F). The dotted red lines represent correlation lines. Pearson's correlation coefficients were used to calculate the correlations, *r* square parameter, and statistical significance (*p < 0.05) is reported on each graph.

Given the observation of a highly heterogeneous distribution of percentages of lymphocytes expressing both ICs and Trm markers (Figure 2), we evaluated the correlation between these parameters and overall survival (OS) in both W‐L and 5‐ALA cohorts. Regarding ICs markers, in the W‐L group, we observed a nonstatistically significant slight inverse correlation between the frequency of CD4+PD‐1+ T cells in GBM and the patient's OS (Figure S4A), that was more evident and near to statistical significance (*p* = 0.06) considering only PD‐1 single positive cells (PD‐1+TIGIT–) (Figure S4B). No correlation with OS was observed for total TIGIT expression in CD4+ T cells and for the other subpopulations identified by PD‐1 and TIGIT expression, neither for both ICs in CD8+ T cells (data not shown). Interestingly, the percentage of total CD4+ T cells expressing PD‐1 as well as of CD4+ T cells PD‐1+TIGIT‐ in the GBM directly significantly correlates with that in the peripheral blood (Figure 5A,B). In the 5‐ALA cohort, no correlation was found between ICs expression in each one of the three analyzed layers with OS as well as with PB (data not shown).

Regarding the expression of the Trm marker CD103, no correlation was observed in the W‐L cohort, both for CD4+ and CD8+ T cells (data not shown). Nevertheless, analyzing different tumor layers obtained with 5‐ALA‐assisted surgery, we observed a statistically significant positive correlation between OS and the percentage of CD4+CD103+ (*p* = 0.038) and CD8+CD103+ (*p* = 0.015) T cells in the necrotic core (Figure 5C,D). No correlation was found between CD103 and OS in the intermediate and marginal tumor layer (data not shown). In addition, there was no correlation between the expression levels of Trm markers in the PB and each of the tumor areas analyzed.

Notably, evaluating the correlation between OS and cytokines production in both W‐L and 5‐ALA cohort, we observed a negative statistically significant correlation between the percentage of CD8+TNF‐α+ T cells and OS in W‐L GBM (*p* = 0.042) (Figure 5E). The same correlation was found also in the CD8+TNF‐α+ T cell of the intermediate area in the 5‐ALA cohort (*p* = 0.034) (Figure 5F). No correlation was found between TNF‐α expression and OS in the other tumor layer and in the PB of both the W‐L and 5‐ALA cohort (data not shown). Regarding the other analyzed cytokine, no correlation with OS was observed in both patient cohorts (data not shown).

## 4 Discussion

According to the WHO Classification 2021, Glioblastoma is categorized as a grade IV tumor, with specific molecular alterations (IDH‐wildtype, *TERT*, *EGFR*, chromosome +7/−1) [15]. It represents the most common and aggressive primary malignant central nervous system tumor in adults [16], whose prognosis is still very poor, despite having many therapeutic strategies [17].

In this study, we provided a phenotypic and functional characterization of immune cells in patients with glioblastoma, with the opportunity to finely analyze TILs obtained from different tumor areas with different metabolic activity thanks to 5‐ALA assisted surgery.

5‐ALA‐assisted surgery for high‐grade gliomas (HGGs) is based on the administration of 5‐ALA orally to patients, before surgery, which leads to intracellular accumulation of fluorescent porphyrin [18]. It was approved by the European Medicines Agency in 2007 and the Food and Drug Administration in 2017 for grades III or IV on the basis of previous clinical studies that determined its safety and utility in discriminating tumor margins during HGG resection [19]. Moreover, it can be associated with functional mapping and improve the removal of HGGs in eloquent areas, increasing the extent of resection (EOR), which correlates with overall survival (OS) and progression‐free survival (PFS) [20]. In our series, OS was lower for patients operated on with 5ALA than under W‐L, since the pathology was spread more diffusely in the brain parenchyma, with major infiltration of subcortical pathways, and the prognosis was poorer. Histological comparison between MRI and 5‐ALA fluorescence‐collected GBM biopsies revealed a stratification of GBM, according to different pink nuances during tumor dissection, with specific characteristics: tumoral necrosis, non‐fluorescent; contrast‐enhancing tumor, bright fluorescent; peritumoral infiltration, visible in FLAIR sequences, vague fluorescent [21].

The frequency of T lymphocyte subpopulations, in particular CD4+ and CD8+ T cells, was first evaluated, given that there are controversial studies about the prognostic significance of TILS in GBM [22, 23]. In our study, in tumor biopsy obtained in white light, we observed a higher frequency of CD8+ T lymphocytes with respect to PB. Interestingly, with the aid of 5‐ALA, this enrichment was found to be mainly confined to the tumor margin. The result seems to be in line with several studies [24], suggesting preferential recruitment of cytotoxic anti‐tumoral CD8+ T cells within the tumor microenvironment and underline the importance of the application of 5‐ALA assisted surgery in order to identify different tumor layers with different immune infiltrates characteristics. Nevertheless, obtained results are based on T‐cell frequencies, additional studies on T‐cell absolute numbers in brain tumors are required to confirm these data.

The presence of Trm cells in many solid tumors correlates with a better prognosis [25, 26, 27], while GBM data present in the literature are not clear. To better elucidate this aspect, we analyzed on both CD4+ and CD8+ T cells the expression of two main markers used for the identification of tissue‐resident memory cells, CD69 and CD103. Our work showed a higher presence of Trm in the tumor mass compared with PB and in particular, these cells seemed to accumulate in the intermediate and margin layers of the tumor suggesting an active recruitment of resident cells in the metabolic active tumoral part, while they are limited in the necrotic core. The prominent presence of Trm within the tumor sample rather than in the PB, is in line with their residency nature and with several studies that underline how Trm cells play a role in cancer immunosurveillance [28, 29]. Interestingly, the correlation of biological data with clinical progression highlights a strongly positive correlation between the frequency of CD103 positive CD4+ and CD8+ T cells in the necrotic core and patients’ OS. This observation agrees with a positive role in antitumor immunity of Trm cells and their correlation with the patient's OS in other solid tumors [25, 26, 30]. Notably, the necrotic core in our cohort showed a lower frequency of CD103 positive T cells compared with intermediate and marginal areas but anyway relevant and higher than PB. Further study on a wider cohort is essential to confirm these data also from a mechanistic point of view.

Along with tissue residency phenotype, also the immune‐checkpoint expression on tumor‐infiltrating lymphocytes could represent potential useful information to be correlated to disease progression.

PD‐1 is one of the most described immune checkpoints in tumor‐infiltrating T lymphocytes and it has been shown to be involved in tumor escape mechanisms [31, 32]. A higher expression of these molecules was already described in GBM samples and our results are in accordance with previously published articles [33]. Nevertheless, less was known about the co‐expression of PD‐1 together with other ICs and no data reported the functionality of PD‐1 expressing T cells. Recent studies have shown that human and murine GBM infiltrating T cells also express a high level of the inhibitory immune‐checkpoint TIGIT [34]. Contrasting results were reported about its correlation with the patient's OS and immunotherapy efficacy [35, 36]. In our study, the frequency of PD‐1 positive cells in the tumor sample is significantly higher than in the PB whereas for TIGIT only a trend was observed without reaching statistical significance. The higher expression of PD‐1 was not related to a particular tumor area but was elevated in all the layers obtained by 5‐ALA, increasing from the Core toward the intermediate and margin layers.

Correlation of biological data with clinical progression highlights, in W‐L patients, a slight inverse relationship between PD‐1 expression in CD4+ infiltrating T cells and patients’ OS. This observation agrees with the immunosuppressive role of PD‐1 [37, 38] and with data from several studies reporting an inverse correlation between ICs expression, in particular for PD‐1, and OS in other solid cancers [39]. Interestingly, the levels of PD‐1 single positive cell frequencies in GBM strongly correlate with those found in the periphery, suggesting that PD‐1 is a marker that could be monitored over time in GBM‐affected patients by fast and low invasive analysis on peripheral blood samples. Nevertheless, these data were obtained in a restricted cohort of patients and in a limited follow‐up time, further information and in‐depth analysis are needed to confirm the results.

To evaluate if the ICs expressing T cells, enriched in the GBM, were actually “exhausted” T cells, we evaluated their cytokine production among total CD4+ and CD8+ T lymphocytes, after ex‐vivo polyclonal stimulation. Interestingly, both tumor‐derived CD4+ and CD8+ T cells produce considerably more TNF‐α and IFN‐γ than those in the PB. These results are also confirmed in biopsies obtained by 5‐ALA. In particular, with regard to IFN‐γ, its production is predominant in the Intermediate layer compared with the Core and Margin. Interestingly, IFN‐γ and TNF‐α are two of the primary cytokines produced by Trm cells, whose markers are enriched in our GBM samples [25]. These data highlight that, although the percentage of CD4+ T cells in the tumor is low and express high levels of ICs, these cells could be efficiently reactivated ex vivo and produce high levels of proinflammatory cytokines: this evidence suggests an antitumor role of CD4+ T cells in GBM microenvironment.

For IL‐17 and IL‐10 there are no differences between PB and total tumor resection. Interestingly, analyzing the different layers we see a not statistically significant trend in a lower expression of IL‐17 in the Intermediate areas while for IL‐10, in particular in CD4+ lymphocytes, there is a statistically significant increase of IL‐10 production in the three tumor layers, compared with PB, with a slight but not statistically significant increment in the Intermediate tumor areas. Together with TGF‐β, IL‐10 is a key immunosuppressive cytokine that is often produced by regulatory T cells. Its slight increase found in the tumor could suggest the presence of Treg lymphocytes within the tumor infiltrate. In particular, the presence of IL‐10‐producing cells in the Intermediate layer suggests that, in the metabolic active tumor, lymphocytes are recruited and are able to produce proinflammatory cytokines such as IFN‐γ, but at the same time some cells produce IL‐10 that could interfere with anti‐tumor activity of IFN‐γ. In addition, our data highlight that in this tumor layer, there is a slight but not statistically significant reduction of IL‐17 production, which is proposed to have an antitumor activity [40], suggesting an immune suppression of TILs, in particular of Th17 and Th1/17 like cells in the intermediate layer.

Cytokine production was evaluated also in the four subsets identified based on PD‐1/TIGIT combinatorial expression. Results revealed that TIGIT single‐positive cells, identify the population of CD4+ lymphocytes that is most exhausted in terms of cytokine production. Unexpectedly, IFN‐γ, TNF‐α, and GM‐CSF are highly produced by CD4+ cells with double positives and single positives for PD‐1. These data agree with the results reported by us in the context of chronic inflammation [41]. In the case of CD8+ lymphocytes, cytokine production is almost homogeneous among the four subpopulations, nevertheless also in this case the PD‐1 positive population produces the highest levels of the proinflammatory cytokines IFN‐γ.

Several studies suggest that a high TIGIT expression correlates with a decrease in cytokines produced by TILs supporting the idea that the most tired population turns out to be the single TIGIT positive [42, 43]. Moreover, in mice and humans, it was observed that tumor‐associated CD103+CD8+ Trm cells maintained increased function and proliferative capacity despite the marked expression of inhibitory immuno‐checkpoints, classically associated with T‐cell dysfunction. This suggests that Trm cells may be more resistant to exhaustion than circulating T cells [44]. Accordingly, our results demonstrate that the CD103+CD69+ lymphocyte population (Trm cells) is widely present at the tumor level, and, in contrast to what we would expect, even double‐positive or single PD‐1+ subpopulations have a very high percentage of proinflammatory cytokine production.

Interestingly, evaluating a possible correlation between cell functionality, in terms of cytokine production, and OS we observed that the frequency of CD8+ T cells expressing TNF‐α negatively correlates with the patient's OS both in the W‐L GBM and in the 5‐ALA intermediate area. Controversial results are reported in several studies about the role of TNF‐α in different solid tumors including GBM [45, 46, 47, 48]. In particular, recent studies show that GBM resistance to antiangiogenic therapy is associated with high levels of TNF‐α in the TME [48]. Indeed, TNF‐α increases the recruitment of immunosuppressive macrophage to the tumor area [48]. In addition, other studies suggest that cytotoxic T cells’ production of proinflammatory cytokines such as IFN‐γ and TNF‐α can trigger upregulation of genes that mediate apoptosis resistance and increase tumor cell proliferation and invasion in GBM cells [49, 50]. Additional data are needed to better clarify the mechanism of action of TNF‐α in the GBM microenvironment. However, our and others’ data corroborate the hypothesis that TNF‐α is a possible novel therapeutic option for GBM patients and future clinical studies should be aimed at inhibiting TNF‐α as a concurrent immunotherapy in GBMs.

In conclusion, this study profiled T‐cell features in GBM showing that different tumor layers were enriched in TILs with different phenotypes and functional properties in terms of cytokines expression. In particular, intermediate and marginal areas were enriched in Trm‐like T cells expressing PD‐1, which, of note, retain a good effector function in terms of cytokines production, whereas TIGIT‐expressing cells produce lower levels of cytokines and were not enriched in GBM. Notably, the frequency of Trm‐like cells in the necrotic core positively correlates with a favorable prognosis, even if this layer results in the tumor portion with the lowest T cells’ infiltrate. In addition, whereas CD8+ T cells produce a high level of the proinflammatory cytokine TNF‐α, the presence of these cells (CD8+ TNF‐α+ T cells) in the GBM intermediate layer negatively correlates with OS, suggesting that TNF‐α should be considered as a new therapeutic option for GBM patients.

These results highlight the relevance of a comprehensive analysis of GBM immune infiltrate to better understand antitumor immune response and to identify new biomarkers, prognostic factors, immunotherapy targets, and guide/support surgery particle strategy considering that tumor areas resected by W‐L surgery are not strictly overlapped with the three areas together of the 5‐ALA assisted one but it mainly corresponds to the core and intense area.

## 5 Data Limitations and Prospectives

Although our findings demonstrate that TILs obtained from different GBM layers showed different phenotypes and functional properties in terms of cytokines expression, several questions remain. Prominent among them is to mechanistically validate our funding on the possible prognostic role of CD103‐expressing cells. In addition, T cells functionality assessed as cytotoxic capacity and not only as cytokine expression should be interesting data to asses. Moreover, additional studies on T cells’ absolute numbers in brain tumors could support obtained results on T cells’ frequencies.

Further studies on GBM tumor samples obtained by 5‐ALA‐assisted surgery integrating obtained T‐cell data with functional studies, also considering T cells’ interaction with myeloid and tumor cells, potentially could lead to identifying new prognostic factors and guide/support surgery particle strategy.

## Author Contributions

Francesco Annunziato and Alessandro Della Puppa designed the study. Alessandro Della Puppa, Camilla Bonaudo, Mirko Petti, and Federico Capelli collected peripheral blood and tumor samples and informed consent. Francesco Liotta and Lorenzo Cosmi provided advice. Anna Vanni, Francesca Matani, Lucia Bartoli, Manuela Capone, Alessio Mazzoni, Laura Maggi, Lorenzo Salvati, Giulia Lamacchia, Filippo Nozzoli, and Stefania Francalanci performed experiments. Anna Vanni and Francesca Matani analyzed the data. Laura Maggi, Anna Vanni, and Francesco Annunziato wrote the manuscript. All authors revised the manuscript and gave final approval.

## Ethics Statement

The procedures followed in the study were approved by the Careggi University Hospital Ethical Committee (protocol code NOI_nch ID15636).

## Conflicts of Interest

The authors declare no conflicts of interest.

## Peer Review

The peer review history for this article is available at https://publons.com/publon/10.1002/eji.202451681.

## Supporting information




**Supporting information file 1**: eji5998‐sup‐0001‐SuppMat.pdf

## Data Availability

The data supporting the findings of this study are available from the corresponding author upon reasonable request.
