# Supplementary material for: 5‐ALA Assisted Surgery of Human Glioblastoma Samples Reveals an Enrichment of T Cells Expressing PD‐1 and CD103 in the Intermediate and Marginal Layers
Source: Eur J Immunol. 2025 Jun 11;55(6):e51681. doi: 10.1002/eji.202451681 (PMC12154168; doi:10.1002/eji.202451681)
Supplement: Supplementary file 1 — Supporting information file 1: eji5998‐sup‐0001‐SuppMat.pdf [file EJI-55-e51681-s001.pdf]

SUPPLEMENTARY TABLES

Supplementary Table 1. Demographic and clinical data of study subjects.

	GENDER	AGE	Histological Features	MGMT methylation (%) *	IDH1 status	OS (months)
**W-L 1**	M	70	Left frontal lobe GBM	1	Wilde type	56 (alive)
**W-L 2**	M	59	Left temporal lobe GBM	1	Wilde type	56 (alive)
**W-L 3**	F	56	Left temporal lobe GBM	8	Wilde type	54 (alive)
**W-L 4**	F	50	GBM not specified	26	Wilde type	53 (alive)
**W-L 5**	F	56	Right parietal lobe GBM	NA	Wilde type	54 (alive)
**W-L 6**	M	46	Right temporal lobe GBM	2	Wilde type	10
**W-L 7**	M	78	Right frontal lobe GBM	1	Wilde type	2
**W-L 8**	F	52	Right temporal lobe GBM	18	Wilde type	36
**W-L 9**	F	57	Left temporal lobe GBM	5	Wilde type	20
**W-L 10**	M	81	Left frontal lobe GBM	51	Wilde type	39 (alive)
**W-L 11**	F	57	Left parietal lobe GBM	3	Wilde type	24
**W-L 12**	M	65	Left temporal lobe GBM	1	Wilde type	1
**W-L 13**	F	64	GBM not specified	5	Wilde type	1
**W-L 14**	M	60	GBM not specified	1	Wilde type	11
**W-L 15**	F	44	GBM not specified	4	Wilde type	13
**W-L 16**	M	60	GBM not specified	3	Wilde type	15 (alive)
**W-L 17**	F	68	GBM not specified	NA	Wilde type	8
**Mean (range)**	-	60 (44-81)	-	8,7 (1-51)	-	26,6 (1-56)
**median**	-	58	-	3	-	20
**SD**	-	10,24	-	13,71	-	21,39
**Male/Female (F/M)**	0,88	-	-	-	-	-
	**GENDER**	**AGE**	**Histological Features**	**MGMT methylation (%) ***	**IDH1 status**	**OS (months)**
**5-ALA 1**	M	79	Right frontal lobe GBM	13	Wilde type	6
**5-ALA 2**	F	82	Right insular lobe GBM	9	Wilde type	6
**5-ALA 3**	M	80	Left temporal lobe GBM	37	Wilde type	24

**5-ALA 4**	M	53	Left temporal lobe GBM	14	Wilde type	37 (alive)
**5-ALA 5**	M	61	Left parietal lobe GBM	2	Wilde type	3
**5-ALA 6**	M	72	Left frontal lobe GBM	2	Wilde type	4
**5-ALA 7**	M	75	Right frontal lobe GBM	44	Wilde type	4
**5-ALA 8**	M	64	Left thalamus GBM	6	Wilde type	3
**5-ALA 9**	M	75	Right Frontal lobe GBM	4	Wilde type	2
**5-ALA 10**	M	73	Right temporal lobe GBM	2	Wilde type	15
**5-ALA 11**	F	64	Left temporal lobe GBM	3	Wilde type	18
**5-ALA 12**	F	54	Right thalamus GBM	51	Wilde type	2
**5-ALA 13**	M	57	Right temporal lobe GBM	7	Wilde type	9
**5-ALA 14**	M	63	Right frontal lobe GBM	1	Wilde type	14
**5-ALA 15**	M	53	Left frontal lobe GBM	54	Wilde type	15
**5-ALA 16**	F	59	Left temporal lobe GBM	NA	Wilde type	18
**5-ALA 17**	F	67	GBM not specified	NA	Wilde type	12
**Mean (range)**	-	66,5 (53-82)	-	16,6 (1-54)	-	11,3 (2-37)
**median**	-	64	-	7	-	9
**SD**	-	9,74	-	19,38	-	9,44
**Male/Female (F/M)**	2,4	-	-	-	-	-

W-L: White light

OS: Overall Survival

IDH1: Isocitrate dehydrogenase 1l

*Percentage of MGMT (O6-Methylguanine-DNA-methyltransferase) promoter methylation

Supplementary Table S2. List of all fluorochrome mAbs used for flow cytometric immunophenotyping of circulating and GBM T cells.

Antigen	Fluorochrome	Clone	Company
CD103	FITC	Ber ACT8	BDBioscience
CD69	PE	L78	BDBioscience
CD3	Super Bright 702	UCTH1	eBioscience™
PD-1	Brilliant Violet 421	EH12.2H7	BioLegend
CD8	APC-Cy7	SK1	BDBioscience
TIGIT	APC	MBSA43	eBioscience™
CD4	PerCP	SK3	BDBioscience
CD161	PE-Vio770	191B8	Miltenyi Biotec

Supplementary Table S3. List of all fluorochrome mAbs used for flow cytometric evaluation of T cells cytokine production in total GBM resection.

Antigen	Fluorochrome	Clone	Company
IFNγ	FITC	25723.11	BDBioscience
IL10	PE	JES3-9D7	Milteniy Biotec
TNFα	PE	6401.1111	BDBioscience
GM-CSF	PE	BVD2-21C11	BDBioscience
IL17	PerCP-Cy5.5	eBio64DEC17	eBioscience™
CD3	Super Bright 702	UCTH1	eBioscience™
CD8	Super Bright 600	SK1	eBioscience™
TIGIT	APC	MBSA43	eBioscience™
CD4	PE-Cy7	SK3	eBioscience™
PD-1	Pacific Blue	EH12.2H7	BioLegend
L/D	Fixable Viability Stain 780		BDBioscience

Supplementary Table S4. List of all fluorochrome mAbs used for flow cytometric evaluation of T cells cytokine production in GBM layers obtained with 5-ALA assisted surgery.

Antigen	Fluorochrome	Clone	Company
IFNγ	Pacific Blue	B27	BioLegend
TNFα	FITC	6401.1111	BDBioscience
IL17	PerCP-Cy5.5	eBio64DEC17	eBioscience™
IL10	PE	JES3-9D7	Milteniy Biotec
CD3	Super Bright 702	UCTH1	eBioscience™
CD8	Super Bright 600	SK1	eBioscience™
CD4	PE-Cy7	SK3	eBioscience™
L/D	Fixable Viability Stain 780		BDBioscience

SUPPLEMENTARY FIGURES

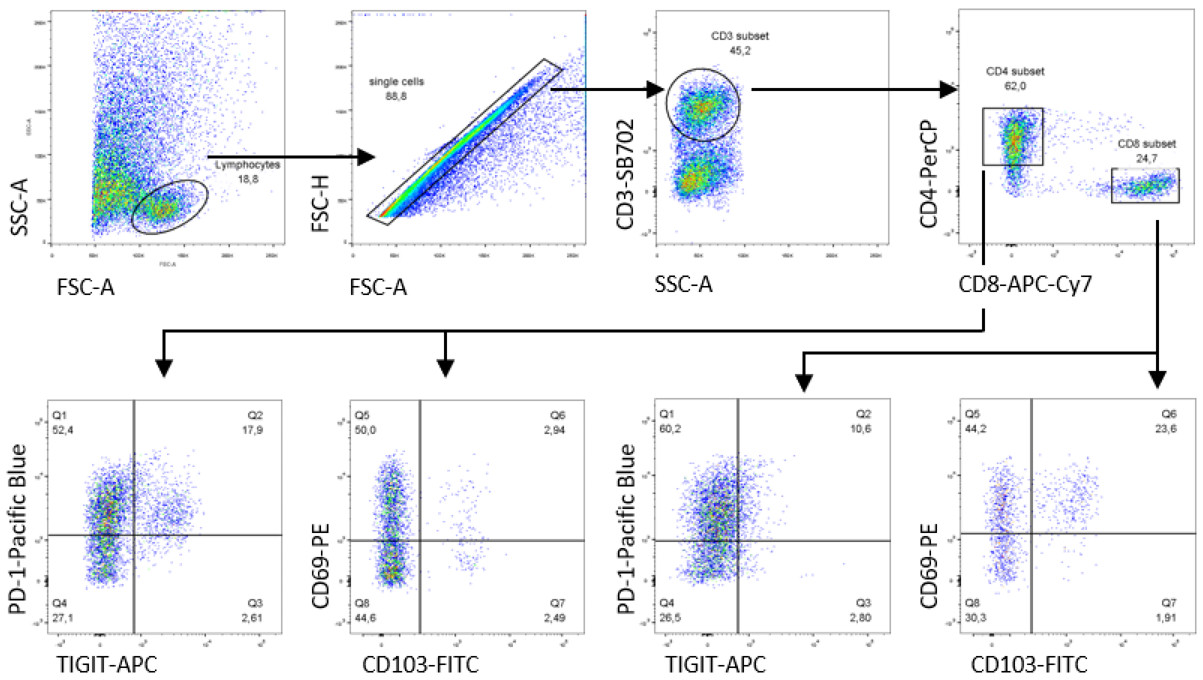

Supplementary Figure 1. Gating strategy used to identify CD4+ and CD8+T lymphocyte expressing PD-1, TIGIT, CD69 and CD103, surface markers. Lymphocytes were gated based on physical parameters (FSC-SSC), then doublets were removed using FSC-A and FSC-H parameters. Based on CD3 expression, CD3+ T cells are identified. Among CD3+ T cells we identified T helper and T cytotoxic lymphocyte based on the expression of CD4 and CD8 marker respectively. Additional subpopulations of both CD4+ and CD8+ lymphocyte populations were found based on variations in the expression of the four surface markers PD-1, TIGIT, CD69 and CD103.

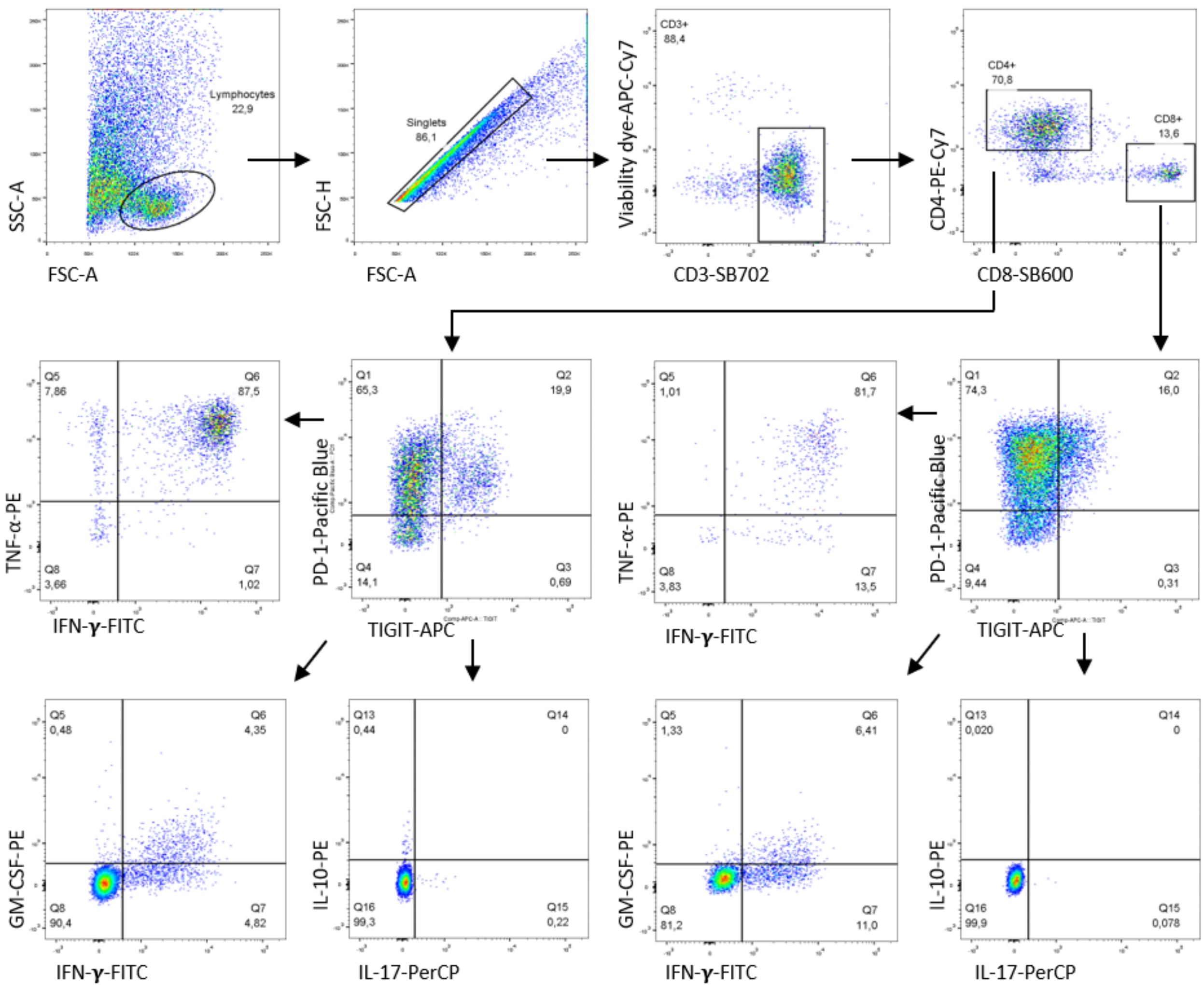

Supplementary Figure 2. Gating strategy for the flow cytometric identification of PD-1 and TIGIT expressing T cells and their differential cytokines production in PB and GBM. Lymphocytes were gated based on physical parameters (FSC-SSC), then doublets were removed using FSC-A and FSC-H parameters. Dead cells were excluded using viability stain and T cells were identified as CD3+. We then identified CD8+ and CD4+ T cells and on both these populations we evaluated TIGIT and PD-1 expression. On the four subpopulations identified based on TIGIT and PD-1 expression (PD-1+TIFIT-, PD-1+TIGIT+, PD-1-TIGIT+, PD-1-TIGIT-) we evaluated the production of IFN-γ, TNF-α, GM-CSF, IL-17, IL-10. TNF-α, GM-CSF and IL-10 are conjugated with the same fluorochrome PE, indeed three different staining were performed in order to evaluate all the listed cytokines.

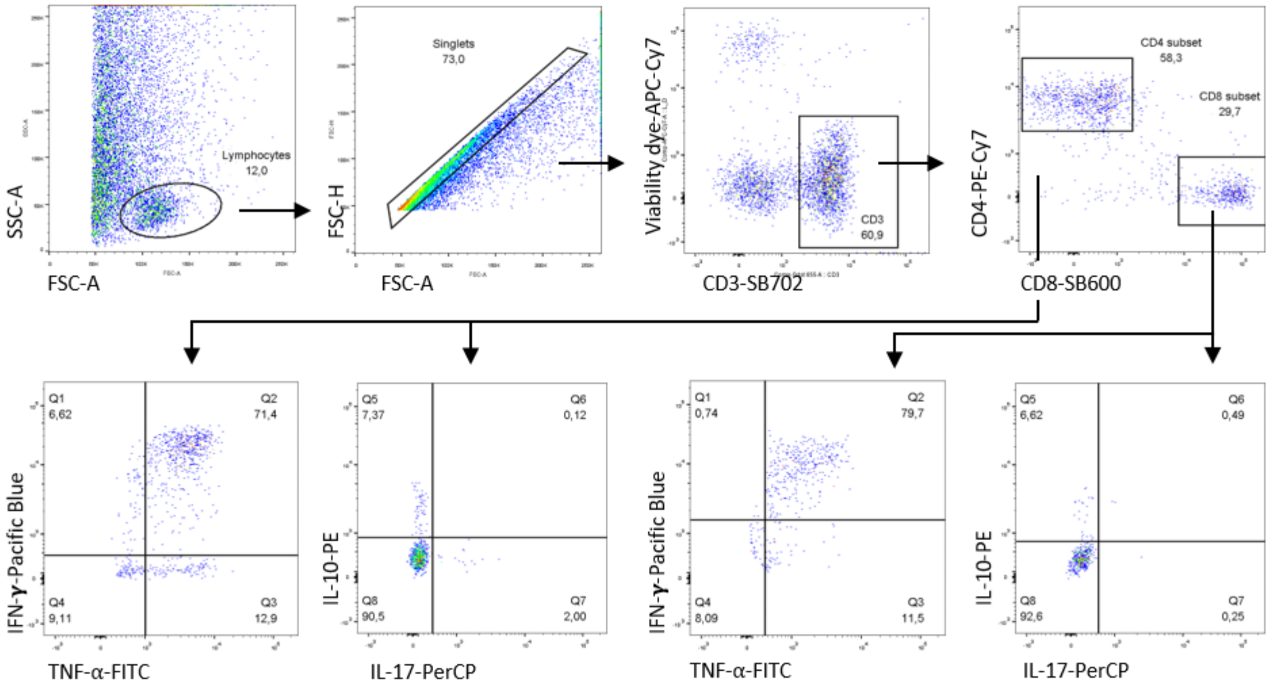

Supplementary Figure 3. Gating strategy used to identify CD4⁺ and CD8⁺ T lymphocyte subpopulations based on the different expression of TNF-α, IFN-γ, IL-10 and IL-17 cytokines in GBM samples obtained with 5-ALA assisted surgery. Lymphocytes were gated based on physical parameters (FSC-SSC), then doublets were removed using FSC-A and FSC-H parameters. Dead cells were excluded using viability stain and T cells were identified as CD3+. The CD3+ population is subsequently separated into $CD8^+$ and $CD4^+$ T cells. For both $CD4^+$ and $CD8^+$ T cells the expression of IFN-γ, TNF-α, IL-10 and IL-17 cytokines was evaluated.

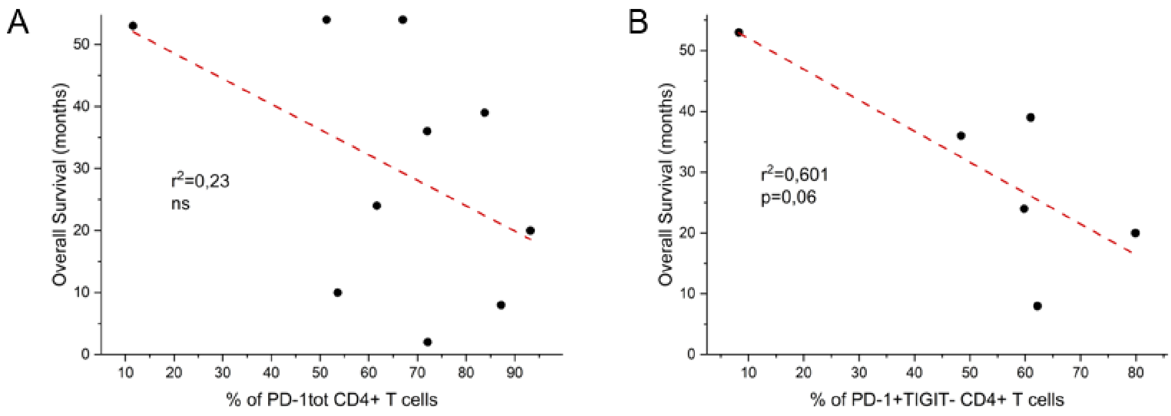

Supplementary Figure 4. Correlation between patient's Overall Survival and GBM CD4+ T cells expressing PD-1. Correlation between percentage of total PD-1 (n=7) (A) or PD-1+TIGIT- (n=5) (B) positive CD4+ T cells, obtained from tumor resection in W-L, and patients' overall survival. Dotted red lines represent correlation lines. Pearson's correlation coefficients were used to calculate the correlations, r square parameter and statistical significance is reported on each graph.
